# HLA-G/C, miRNAs, and Their Role in HIV Infection and Replication

**DOI:** 10.1155/2013/693643

**Published:** 2013-06-13

**Authors:** Fulvio Celsi, Eulalia Catamo, Giulio Kleiner, Paola Maura Tricarico, Josef Vuch, Sergio Crovella

**Affiliations:** ^1^Institute for Maternal and Child Health, IRCCS Burlo Garofolo, 34137 Trieste, Italy; ^2^University of Trieste, 34128 Trieste, Italy

## Abstract

In recent years, a number of different mechanisms regulating gene expressions, either in normal or in pathological conditions, have been discovered. This review aims to highlight some of the regulatory pathways involved during the HIV-1 infection and disease progression, focusing on the novel discovered microRNAs (miRNAs) and their relation with immune system's agents. Human leukocyte antigen (HLA) family of proteins plays a key role because it is a crucial modulator of the immune response; here we will examine recent findings, centering especially on HLA-C and -G, novel players lately discovered to engage in modulation of immune system. We hope to provide novel perspectives useful to find out original therapeutic roads against HIV-1 infection and AIDS progression.

## 1. Introduction

Gene expression is a tightly regulated mechanism, in a cell- as well as time-specific manner, and numerous different pathways exist to regulate this activity. Human immunodeficiency virus (HIV) infection is able to perturb and alter gene expression through several mechanisms that can, lastly, cause acquired immunodeficiency syndrome (AIDS). In this review, we will focus on novel mechanisms of gene expression regulation, centering on recently discovered players in the interaction between HIV and immune system. Major histocompatibility complex class I molecules (MHC-I) are necessary for an efficient host immune response to HIV-1 infection, as detailed below. A subset of MHC-1 allotypes are associated with effective control of viral replication and slow disease progression. In a series of recent genome-wide association studies, a relevant association between some HLA-C single-nucleotide polymorphisms (SNPs) and HIV-1 infection has been found [1–4], and similar researches have been done also for HLA-G, as detailed below [5, 6].

### 1.1. HIV Infection and Replication

**Figure 1:**
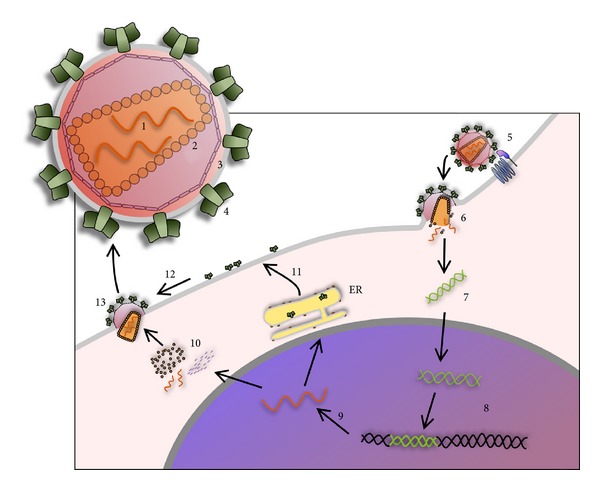
HIV virus is formed by a diploid single strand RNA genome (1) enclosed in a truncated cone capsid (2) with a phospholipidic bilayer envelope (3), containing the proteins that allow the virus entry into the cells (4). The HIV-1 infection is mediated by interaction between the proteins of the viral envelope, leukocyte receptor, and coreceptor (5). This interaction causes the membranes fusion and the uncoating of the virion core (6). The viral RNA is reverse transcribed in DNA (7) which enters in the nucleus where the integrase enzyme catalyzes the insertion of the viral genome into the genome of the host cell (8). The expression of integrated viral genome is controlled by the RNA-binding proteins *tat* and *rev*. A set of RNAs are transported from the nucleus to the cytoplasm, where they can be translated or packaged (9). The new core proteins localize near the cell membrane (10), while the envelope mRNA is translated at the endoplasmic reticulum (ER) and subsequently the envelope proteins are placed on the cell membrane (11). Finally, the capsid proteins are assembled with the viral genomic RNA (12), and an immature virion begins to bud from cell surface (13).

The human immunodeficiency virus (HIV) is an RNA virus included in the genus *Lentivirus*, family Retroviridae, and it is the cause of the AIDS. This virus is formed of a diploid single strand RNA genome enclosed in a truncated cone capsid with a phospholipidic bilayer envelope, containing the proteins that allow the virus entry into the cells. The HIV-1 genome consists of three sequences,* gag*, *pol, *and *env*.* Gag* codes for the proteins, p6 of the viral capsid, p7 of the nucleocapsid, and p17 of the matrix. *Pol *codes for the replicative enzymes required for the biological cycle and replication, as reverse transcriptase, protease, and so forth. Finally, *env* codes for gp160, which is the precursor of the viral envelope proteins, gp120 and gp41. Furthermore, the HIV-1 genome present 6 genes encoding proteins that regulate the life cycle of the virus [7], as *tat* and *rev*, essential for replication, *vif*, *vpu, *and *vpr*, which regulate the ability of replication and assembly of the new viral particles, *nef*, which reduces the expression of CD4 on the host cell and promotes virus release [8]. The HIV-1 infection is mediated by interaction between the proteins of the viral envelope, gp120 and gp41, and the receptor of T lymphocyte, CD4. This interaction causes a conformational change of gp120 that binds the coreceptors CXCR4 and CCR5, present on the surface of T lymphocyte [9]. A hydrophobic region of gp41, called “fusion peptide,” penetrates the membrane of the target cell and causes the fusion between plasmalemmas. The virion core is then uncoated to expose a viral nucleoprotein complex, and then the RNA of this complex is reverse transcribed in DNA. The viral DNA thus enters in the nucleus together with the integrase enzyme, which catalyzes the insertion of the viral genome into the genome of the host cell (see Figure 1). 

The expression of integrated viral genome is controlled by the RNA-binding proteins, *tat* and *rev*, able to orchestrate complex interactions with the cellular transcription, RNA splicing, and RNA transport machinery. HIV latency arises when levels of the regulatory protein *tat* fall to below threshold levels [10].

A set of RNAs, either spliced or full genome length, is transported from the nucleus to the cytoplasm, where RNAs can be translated or packaged. The new core proteins localize near the cell membrane, while the envelope (*env*) mRNA is translated at the endoplasmic reticulum (ER), and subsequently the envelope proteins are placed on the cell membrane. Finally, the capsid proteins are assembled with the viral genomic RNA, and an immature virion begins to bud from cell surface. In this process the virions are also coated with *env*. Before having the ability to infect another cell, the virion undergoes a morphological change known as maturation that includes proteolytic processing of the Gag and Gag-Pol polyproteins by viral enzyme PR and a less well defined function of Vif [8].

### 1.2. Small Noncoding RNA

The human genome is constituted by protein-coding sequences for only two percent while the remaining vast majority of it, previously considered as “junk DNA,” could be transcribed and it is now named as non-coding RNA (ncRNA). These different RNAs could be responsible for some of the complex differences between humans and other primates, since protein-coding components are extremely similar even in these different species [11]. The ncRNAs present in the cell are the already well-known transfer RNA (tRNA) and ribosomal RNA (rRNA) together with the less-known small nucleolar RNA (snoRNA), long noncoding RNA (lncRNA), piwi-interacting RNA (piRNA), and microRNA (miRNA). SnoRNAs are small molecules of RNA found in the nucleolus: they are responsible for guiding a series of site-specific posttranscriptional modifications in tRNA and rRNA, such as conversion of the nucleoside uridine to pseudouridine [11]. The so-called “long” ncRNAs are over 200 nucleotides in length (sometimes even more than 15 kb) and, in the majority of cases, do not share sequence homology between each other. These longer transcripts are spliced, capped, and polyadenylated, and they are involved in the regulation of the gene expression [11, 12]. PiRNAs are the largest class of small ncRNAs expressed in vertebrates; generally ranging from 25 to 33 nucleotides in length, piRNAs play a key role during spermatogenesis in defending germline cells against transposons by selectively silencing them [11, 13].

Last but not least miRNAs, 22 nt endogenous RNAs, are known to play important regulatory roles in eukaryotic cells by targeting mRNAs for cleavage or translational repression. The first step in the miRNA biogenesis is the transcription by (usually) polymerase II of a long sequence called “primary-miRNA” (pri-miRNA), subsequently cleaved in the nucleus to liberate a 60–70 nt stem loop intermediate, known as the miRNA precursor (pre-miRNA). An RNase III endonuclease, called Drosha, cleaves the RNA duplex with a staggered cut, and thus the base of the pre-miRNA stem loop has a 5′ phosphate and 2 nt 3′ overhang. The next step is the pre-miRNA active transport from the nucleus to the cytoplasm by Ran-GTP and the export receptor Exportin-5. In the cytoplasm, the Dicer enzyme (another RNase III endonuclease) cuts both strands of the duplex and leaves the 5′ phosphate and 2 nt 3′ producing an imperfect duplex. These strands produced by Dicer are incorporated as single-stranded RNAs into a ribonucleoprotein complex, known as the RNA-induced silencing complex (RISC). The RISC identifies target sequences based on Watson-Crick complementarity between the 3′ miRNA and the mRNA.

If the annealing between the sequence loaded on the RISC complex and the target is perfect, then Argonaute protein (specifically Ago2, one of the component of RISC complex) cleaves the RNA duplex approximately in the middle, producing two “naked” RNA sequences (one without the poly-A signal, the other without the “cap”) that are subsequently degraded. If the annealing between the miRNA sequence and the target sequence is not perfect, the Ago2 RNase activity is not present, nonetheless the mRNA translation is inhibited (through not yet clear mechanisms), and the target mRNA is subsequently degraded [11, 14].

### 1.3. Human Leukocyte Antigen

The major histocompatibility complex-1 (MHC-1), also known as human leukocyte antigen-1 (HLA-1), plays a central role in both adaptive and innate immunity; its locus, mapping at chromosome 6 in the human genome, comprises two subgroups, the classical class I molecules (HLA-1a) and the nonclassical molecules (HLA-1b). The highly polymorphic HLA-1a family includes HLA-A, -B, and -C, widely expressed in most tissues, where they expose on the cell surfaces the antigenic peptides. In contrast, the nonclassical HLA-1b family includes HLA-E, -F, and -G: they are less polymorphic and often exhibit a relatively restricted tissue distribution [15].

HLA-1a and -1b molecules consist, respectively, of a 44 kDa and 41 kDa heavy *α*-chain (divided in 3 subdomains: *α*1, *α*2, and *α*3) noncovalently associated with a light β-chain, the β_2_-microglobulin (β_2_M), and a short antigen peptide derived from the degradation of intracellular proteins [16]. In particular, HLA-1a molecules are membrane-bound proteins, while HLA-1b molecules have membrane-bound isoforms and soluble isoforms [17]. 

**Figure 2:**
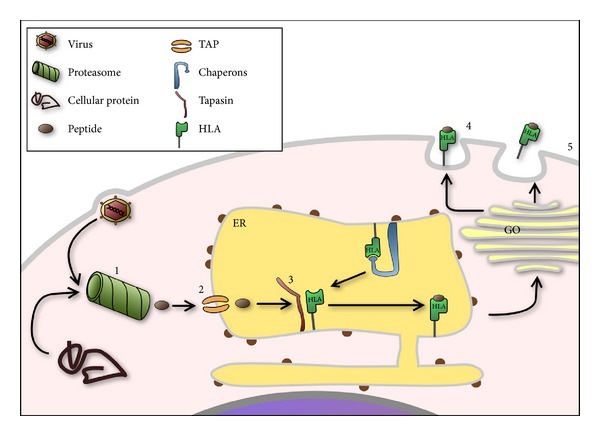
MHC1 are assembled in the lumen of the endoplasmatic reticulum where interaction with target peptides is mediated by chaperons. These peptides, cellular or viral derived, are produced in the cytosol by proteasome (1) and are transported into the ER lumen by TAP pump (2). Subsequently, the chaperon tapasin interacts with TAP, promoting the bond between peptides and HLA (3). The complex HLA peptide egresses through the golgi complex to the plasma membrane where it can remain (4) or can be released in soluble form to the extracellular space (5). ER: endoplasmic reticulum. GO: golgi apparatus.

MHC-1 molecules are assembled in the lumen of the endoplasmic reticulum (ER) with the help of molecular chaperones before being loaded with the immunological-relevant peptides. These peptides are of cellular or viral origin, and they are produced in the cytosol by proteasome. Transporter associated with antigen processing (TAP) pumps the peptides into the ER lumen where they are bounded to MHC-1 thanks to the adaptor protein tapasin. After leaving the ER, the complexes egress through the golgi to the plasma membrane where it can remain or can be released in a soluble form to extracellular space (see Figure 2). In this way, MHC-1 can interact with the T cells and the natural killer cells (NK) [18].

HLA genes are extremely polymorphic; for this reason antigen-presenting cells can differently present a specific antigenic peptide to the immune system. Therefore, HLA genes have also been studied as candidate genes able to modify host's genetic background during infectious diseases progression [19].

#### 1.3.1. HLA-C

HLA-C is naturally expressed on the cell surface at levels approximately 10-fold less than most HLA-A and HLA-B allotypes, and fewer alleles of this protein have been identified, as compared to HLA-A or -B [20]. HLA-C plays a dual role: it presents antigen to cytotoxic T lymphocytes (CTLs), albeit less efficiently than either HLA-A or -B [21], while it results more efficient in inhibiting natural killer (NK) cell lysis via its interaction with inhibitory killer cell immunoglobulin-like receptor (KIR) [22].

Many viruses, such as HCV [23] and HIV [24], use this inhibitory capacity to facilitate their infections in host organism. Indeed, viruses take advantage of several regulatory mechanisms in order to modify levels and distribution of HLA-1a. In all cases, protection from attack by cytotoxic T lymphocytes (CTL) is primarily mediated by down-regulation of HLA-A and -B, but not HLA-C. In this way, the presence of HLA-C may allow inhibition of NK cells expressing KIRs [25, 26]. However, high HLA-C expression levels could damage viral infections due to the increase of the antigen presentation to CTL. For these reasons, the HLA-C levels are finely tuned during viral infections [27].

#### 1.3.2. HLA-G

HLA-G proteins can be expressed as seven distinct isoforms, by means of an alternative splicing from a single primary transcript. Four isoforms are membrane-bound proteins (HLA-G1, -G2, -G3, and -G4), and the other three isoforms are soluble proteins (HLA-G5, -G6, and G7) [17]. HLA-G molecules are not ubiquitously expressed, albeit they are detected under physiological conditions in several tissues, such as placental trophoblast cells, thymus, cornea, nail matrix, pancreas, and some cells of the immune system as monocytes [28, 29]; however, in nonphysiological conditions, (i.e., in tumors) HLA-G molecules are overexpressed in aberrant patterns [30].

HLA-G is an important immunomodulatory molecule, and it plays a fundamental role in maternal-fetal tolerance, transplantation, and in tumor progression [31]. HLA-G molecules can inhibit natural killer (NK) cells lysis and cytotoxic activity of CD8^+^ T cells by interaction with inhibitory receptors KIR, LILRB1, and LILRB2 [32]. The inhibition of both these classes of inflammatory cells creates an anti-inflammatory environment, due to a release of cytokines, such as interleukin 10 (IL-10), with anti-inflammatory properties that are able to upregulate HLA-G, and those in turn increases IL-10 secretion, thus creating a positive-feedback loop [28]. This immunosuppressive capacity of HLA-G is exploited by many viruses, which have developed multiple strategies for subverting host immune defenses. In fact, it has been reported that HLA-G expression in cells is also upregulated following infection with human cytomegalovirus (CMV) and HIV [33, 34].

## 2. HLA and HIV

During viral infection, some viruses undergo degradation by cellular proteasome complex, and the cytosolic antigenic peptides are carried into the ER. In this organelle, the peptides are captured by HLA I molecules and then exposed on the cell surface, triggering the cytotoxic activity of the circulating CD8^+^ T lymphocytes, as described previously.

**Figure 3:**
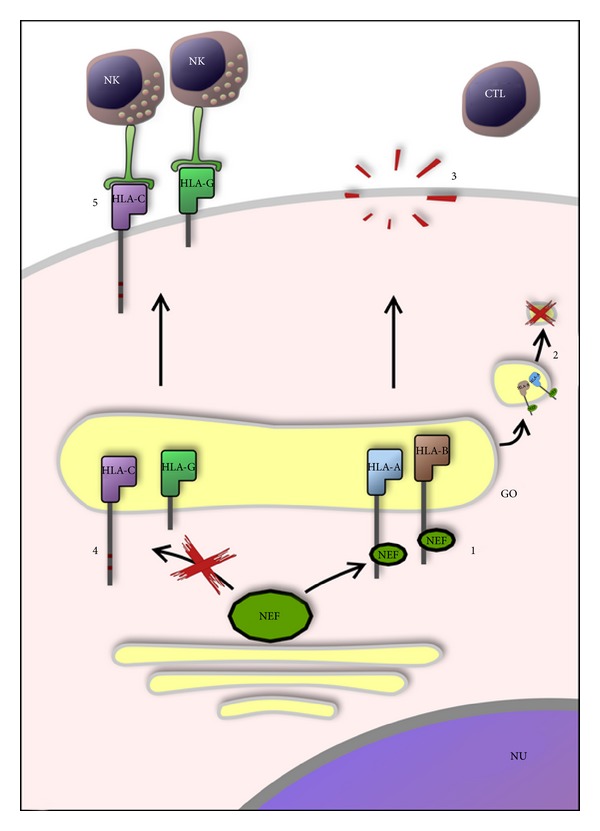
The HIV-1 has developed a nef-dependent mechanism to escape the immune system attack to infected cells. Nef alters the expression of HLA-A and -B by recognition of a sequence (Y_320_SQAASS) in the cytoplasmic tail (1), blocking the transport of newly synthesized HLA-A and -B (2), in order to avoid the recognition by CTL (3) while Nef maintains the expression of HLA-C (C_320_SQAASS) and -G (short cytoplasmic tail) unchanged (4) in order to inhibit the innate response of the NK (5). NU: nucleus; GO: golgi apparatus.

The HIV has devised different ways to evade the immune response, including a Nef-dependent mechanism that downregulates the HLA I expression, thus avoiding the recognition of the infected cells by CD8^+^ T lymphocytes [25]. Selectively, Nef alters the expression of HLA-A and -B by recognition of a sequence (Y_320 _SQAASS) present on the cytoplasmic tail of these HLA molecules [35, 36] accelerating their endocytosis from the plasma membrane [37–39] and blocking the transport of newly synthesized MHC class I molecules to the cell surface [40]. Nef maintains the expression of HLA-C [41] -G, and -E unchanged [42, 43], in order to inhibit the innate response of the natural killer cells (NK) [41] (see also Figure 3).

Furthermore, the gp41 protein of the viral envelope upregulates the synthesis of IL-10 by monocytes [44]; in turn, as mentioned before, this cytokine increases the expression of HLA-G molecules [28] to control immune response and facilitate infection [45]. 

### 2.1. HLA-G and HIV

Numerous studies have been conducted, aimed at observing the expression of the molecule HLA-G in the early stage of infection by HIV and its progression. In 2004, Derrien and colleagues demonstrated that during HIV-1 infection the HLA-G1 isoform was downregulated by a Vpu-dependent mechanism, which recognizes a double lysine residues in 4 and 5 positions of the C terminus [46]. The HLA-G1 isoform has the major ability to present viral peptides to CD8^+^ T lymphocytes [47]; therefore, the recognition of HIV-1 infected cells by CD8^+^ T lymphocytes could depend on the expression of HLA-G1 [46].

In contrast, in 2002, Lozano and collegues observed high surface expression of HLA-G on monocytes and on some T lymphocytes in HIV positive patients with or without antiretroviral treatment. They hypothesized that the high expression of HLA-G was an indirect induction by infection of HIV-1, related to the pathogenesis of the infection, considering that HLA-G expression occurs in a very high proportion of monocytes, which are unlikely to be infected [48]. Another possible explanation, however, is the possibility that this increase is caused indirectly as a consequence to high levels of cytokines, as IL-10 [28]. 

Donaghy et al. observed in 2007 high circulating levels of soluble HLA-G (sHLA-G) in HIV-1-infected individuals, and they hypothesized that these levels could depend on the release of the membrane-bound moiety and thus participate to the pathogenesis of HIV by inducing tolerance [49]. In agreement with the findings by Donaghy and collegues, a subsequent work by Murdaca et al. [50] showed elevated serum levels of sHLA-G in HIV-1-positive individuals. These results were correlated with parameters of immunological and virological response to antiretroviral treatment, and the authors found decreased levels of sHLA-G in patients in which the replication of HIV-1 was suppressed during Highly Active Antiretroviral Therapy (HAART) treatment, while the sHLA-G levels remained elevated in patients in which were present high levels of HIV-RNA after 36 months from antiretroviral therapies. These elevated serum levels of sHLA-G may depend on the increased production of cytokines during the HIV-1 infection, contribute to the immunosuppressive state of the HIV-1-positive individuals, and facilitate their progression to AIDS [50].

Lajoie et al. in 2009 performed a longitudinal study evaluating sHLA-G plasma levels in HIV-1-infected patients with different rates of clinical progression to determine whether sHLA-G expression was associated with HIV-1 infection expansion. The authors observed elevated levels of sHLA-G in the early stages of HIV-1 infection, whereas in the chronic stage, in untreated normal progressor, and in long-term progressor the sHLA-G levels were restored to normality, as a result of the immune system's ability to control the HIV-1 infection. Conversely, the levels of sHLA-G remained high in rapid progressor HIV-1-positive patients. Once again, these data were justified by the high concentration of IL-10 in rapid progressor patients with respect to HIV-1-negative individuals. Another explanation could be the elevated blood levels of mature plasmacytoid dendritic cells, major producers of sHLA-G [51], during the chronic stages of the infection in rapid progressor individuals with respect to HIV-negative subjects [52].

Although myeloid dendritic cells are the major producers of HLA-G, together with plasmacytoid dendritic cells [51], Hazenberg et al. in 2010 demonstrated that elevated levels of sHLA-G mediate the dysfunction of the myeloid dendritic cells through the binding to the myelomonocytic MHC-I receptor ILT4 during the progression of the HIV-1 infection. Therefore, the authors hypothesized that elevated levels of HLA-G may inhibit the antigen-presenting properties of dendritic cells, while they enhance the capacity of the dendritic cells to secrete proinflammatory cytokines, as IL-12p70, determining an important proinflammatory environment for the HIV-1 immunopathogenesis [53].

Lajoie et al. in 2010 observed lower plasma levels of sHLA-G in HIV-1-positive Beninese commercial sex workers, and they hypothesized that the difference of sHLA-G levels could be genetically determined [52]. In agreement with this hypothesis, Turk and coworkers in 2013 reported that the HLA-G*01:01:01 genotype was significantly associated with HIV-1-resistant women, while the HLA-G*01:04:04 genotype was significantly associated with susceptibility to HIV-1 infection [54].

Matte et al. in 2004 demonstrated a highly significant association between HLA-G*0105N allele (null allele) and protection from HIV-1 infection. These authors proposed that this allele, not encoding a functional soluble or membrane-bound HLA-G1 isoform [55], could facilitate the natural killer cells to destroy HIV-1-infected cells [45].

Conversely, Segat and colleagues in 2010 showed a significant increase of HLA-G*0105N allele in HIV-1-positive women patients with respect to HIV-1-negative women controls. They suggested that the different results obtained with respect to the study made by Matte et al. were imputable to the different ethnicity of the studied population. Moreover, they hypothesized that the association between HLA-G*0105N and the increased risk of HIV-1 infection was caused by the capacity of the null allele, when present, to increase the production of other HLA-G isoforms, thus compensating the absence of HLA-G1 and -G5 [56] and inhibiting the NK lysis [5]. 

HLA-G mRNA transcription and translation are tightly regulated processes, giving the immunosuppressive property of this molecule. Some recent works have discovered that HLA-G 3′UTR mRNA has some binding site for miRNAs. Castelli and coauthors in 2009 have made an extensive *in silico* analysis of the HLA-G 3′UTR region, seeking for putative miRNA binding site. They found that different miRNAs bind to this region and moreover the vast majority of these sequences encompass eight highly polymorphic sites [57]. One of them above all, the C/G polymorphism at position +3142, putative binding site for at least three miRNAs, hsa-mir-148a, hsa-mir-148b, and hsa-mir152, has been subsequently confirmed to be the binding site for mir152, and this binding reduces HLA-G expression [58]. However, the effect of this polymorphism in miRNA binding has been recently questioned by Manaster and colleagues. They confirmed previous results, finding that mir-152 and mir-148a bound to HLA-G 3′UTR and downregulate its mRNA, but they also found that the C/G polymorphism at +3142 has no effect on miRNAs binding and efficacy [59]. Interestingly, they found in placenta low levels of these miRNA, and they suggest that this could be a regulation mechanism, allowing HLA-G expression only where needed and not in other district, where the immunosuppressive activity of this molecule could be detrimental. In an interesting parallel, however, the hsa-mir-148a has been proposed to bind HLA-C 3′UTR, and a polymorphism in the binding site for this miRNA, which increase the binding strength, has been associated with poor HIV-1 infection control (see the following section). Thus, the connection between miRNA, HLA-G expression, and HIV-1 needs to be further explored because it can reveal novel information about HIV-1 control of the immune system. 

### 2.2. HLA-C and HIV

The mechanisms that regulate HLA-C expression and the link between this molecule and HIV infection are not yet completely understood. HLA-C can present antigens to CTL, and it is able to inhibit NK cell lysis, but for some reason it is normally expressed on the cell surface at levels approximately 10-fold less than most HLA-A and HLA-B allotypes [60]. This observation could be explained by a new study, focused on a new miRNA targeting sequences identified on HLA-C gene and regulating the surface expression of the protein (see the following paragraphs) [61]. Another possible explanation for this low level of expression comes from a study focused on the low level of affinity between β2M and the HLA-C heavy chains, which are then accumulated in the ER and ultimately degraded [62].

HIV-1 also is able to regulate via Nef the expression of MHC-I molecules. However, since the removal of class I molecules from the surface of virus-infected cell may result in attacks by NK cells, HIV-1 significantly downregulates HLA-A and HLA-B [25], recognized by the majority of CTL, but not HLA-C and HLA-E, thus maintaining their inhibitory role on NK. This selective regulatory activity allows viruses to counteract at the same moment both innate and adaptive immune responses. This mechanism of class I downregulation is a bypass of the immune response, even if it does not ensure to the virus an unfailing escape from the immune system [41, 63].

Indeed, even the host can count on several processes aimed at protecting itself from viral infection. In the last years several studies focused on HLA-C in affecting HIV disease progression. One single-nucleotide polymorphism (SNP) 35 kb upstream of HLA-C locus (-35C/T) has been shown to be crucial for the HIV progression [3, 64] and the surface expression of HLA-C, with higher surface expression associated with slower disease progression [65]. This two aspects resulted to be strictly linked by functional studies demonstrating that -35C allele leads to higher HLA-C cell surface expression and that AIDS and viremia progress slower in individuals with high-expressing HLA-C alleles compared to individuals with low HLA-C expressing alleles (-35T) [27, 65]. Although the mechanism underlying this changing expression is still unknown, the augmented expression of surface HLA-C mediated by the genetic variant -35C may bypass the regulatory activity of Nef, thus resulting in being particularly protective because HIV-1 does not affect HLA-C, as detailed previously [63]. Moreover, the protective effects of the allele -35C could be exerted through a more effective antigen presentation to CTL or an enhancement of NK cell activity driven by the high levels of HLA-C on the infected-cells surface [27]. Substantially, the -35CC genotype (i.e., the presence of C allele at both strands in -35 position) may improve control of HIV/AIDS thanks to an augmented HLA-C surface expression resulting in an enhanced antigen presentation.

**Figure 4:**
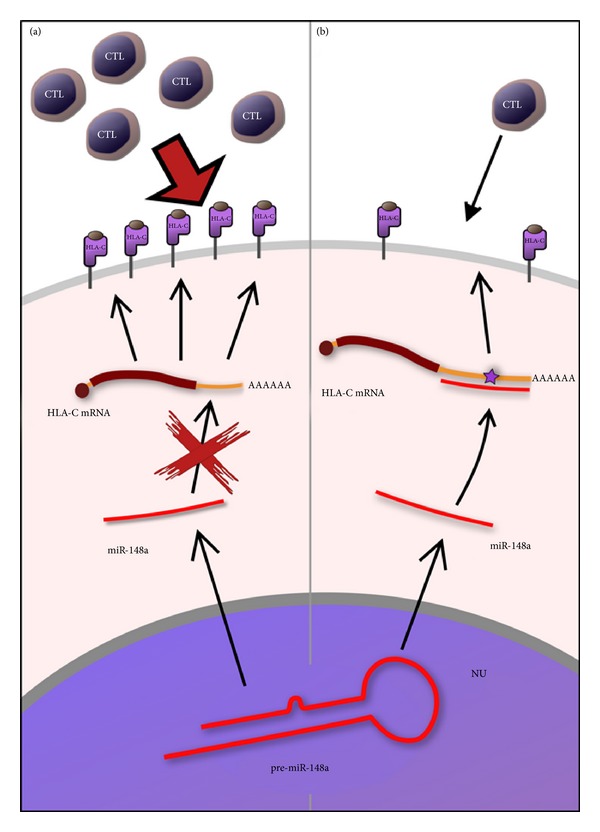
The 263del/ins SNP associates strongly with surface expression of HLA-C. (a) 263del disrupts the binding site of miR-148a not altering the expression of HLA-C. (b) 263insG increases the stability of the binding with the miR-148a reducing surface expression of HLA-C and the recognition by CTL.

However, this protective effect could be not so strong since there are evidences showing that the -35T SNP does not lead to an unequivocal correlation with low HLA-C expression levels because there is an overlap in the distribution of HLA-C surface expression between the different genotypes; moreover, some individuals with the -35CC alleles exhibit high viral loads. The authors thus proposed that the amount of HLA-C, expressed on the cell-infected surface, could represent the protective mechanism itself, rather than the -35C/T genotype [63]. This suggestion means that the -35C/T SNP could not be so fundamental in the control of the HIV progression. Indeed, control of HLA-C surface expression has been correlated with the presence of a microRNA binding site that affects HLA-C expression and the control of HIV disease [61]. It has been suggested that the -35 SNP could not be the real cause for differential HLA-C expression, but rather the marker of another polymorphism, able to directly affect levels of surface HLA-C. In fact, linkage disequilibrium between the -35 SNP and a single nucleotide insertion/deletion at the 3′UTR of the HLA-C gene, directly into the binding site of microRNA hsa-miR-148, has been reported. The 263del/ins SNP associates strongly with the control of HIV-1 replication, since the 263insG increases the stability of the binding with the miR-148a/miR-148b, leading to a low surface expression of HLA-C. On the opposite, 263del disrupts the binding site, leading to a high surface expression of HLA-C (see also Figure 4). The authors found that 263insG is in linkage disequilibrium with -35T allele. These data suggest that 263ins/del could be the causal variant for differential HLA-C expression and subsequently for AIDS/HIV progression.

## 3. miRNA and HIV

### 3.1. HIV Regulates Cellular miRNA

HIV-1 infection could change the intracellular miRNA milieu, as many different viruses do. One of the important characteristics of *Tat* protein, encoded by the viral genome, is its silencing RNA (siRNA) suppressor (SRS) activity. Some reports say that *Tat* interacts directly with Dicer, thus blocking its ability to process miRNA precursors [66]. Others affirm that *Tat* would sequester miRNA duplexes, through a nonsequence-specific mechanism, thus effectively blocking the RISC pathway [67]. This SRS activity is a common mechanism, shared by other viruses (i.e., influenza virus, Ebola virus, and others) to effectively suppress one of main cellular defense from infections (for a review see [68]). 

Apart from this SRS mechanism, HIV-1 infection could change intracellular miRNA response in other ways. In *ex vivo* experiments using lymphocytes, Hayes et al. (2011) found changes in expression of 145 miRNA [69] (and only 22 could be explained by *Tat* SRS activity), and in a subsequent work Sun et al. (2012) found reduced expression of several cellular miRNAs. miR29a, miR29b, and miR29c appear reduced in both these works, thus suggesting a pivotal role for these miRNAs in HIV-1 infection [70]. These studies made in *ex vivo* settings maybe do not correctly depict the real *in vivo* situation. Recent studies, however, start to delineate the *in vivo* situation, a more challenging condition because in infected individual only a small fraction of CD4^+^ T cells are HIV-1 positive (estimated to be 1 in 10000 in blood and 1 in 100 in lymph nodes) [71]. In a seminal work in 2008, Houzet et al. profile miRNA from PBMCs in 36 individuals, classified as patients with high CD4^+^ T cell count and low viral load (class I), high CD4^+^ T cell count and high viral load (class II), low CD4^+^ T cell count and low viral load (class III), and low CD4^+^ T cell count and high viral load (class IV). They found miRNA profiles specific for those different four classes of patients [72]. In a subsequent study, Witwer and coworkers analyzed miRNA profiles from healthy individuals, elite HIV-1 controllers, and untreated viremic HIV-1 patients. Their results confirm and expand Houzet's work, especially data on viremic patients, because they show downregulation of miRNA 150 e miRNA 29 families (two crucial miRNAs in other pathways, as shown below). Moreover, they correlate miRNA changes with clinical parameters, that is, CD4^+^ T cell counts and viral loads, and they found that miRNA expression could be used as a biomarker for HIV-1 disease progression [73]. 

A crucial point to understand how HIV-1 changes miRNA cellular milieu is to define the differences between cells prone to HIV-1 infection, that is, activated CD4^+^ T cells, and cells that are less liable to this infection, that is, resting CD4^+^ T cells and monocytes. A groundbreaking work in this field is the one done by Huang and coworkers in 2007. They found that miR-28, miR-125b, miR-150, miR-223, and miR-382 are enriched in resting CD4^+^ T cells. Those miRNAs have binding site at the 3′UTR HIV-1 sequence and thus, upon binding, repress viral RNA expression. According to the authors, this mechanism could explain HIV-1 latency in resting CD4^+^ T cells [74]. Another important miRNA in regulating HIV-1 expression is miR-29a, that has been found to be highly expressed in resting CD4^+^ T cells and, more importantly, to be able to target both the 3′ end of HIV-1 transcript and the HIV-1 encoded protein Nef, thus interfering with HIV-1 expression and replication [75]. Moreover a recent report confirms these previous results, using resting and activated CD4^+^ T cells. Chiang and coworkers identify miR-27b, miR-29b, miR-150, and miR-223 as being significantly downregulated upon CD4^+^ T cell activation. MiR27b binds to, among others target, cyclin T1 3′UTR. This protein is crucial for HIV-1 replication process, as the viral *Tat* recruits and binds it to promote viral genome transcription. The authors thus suggest that miR27b could directly regulate HIV-1 transcription, while the other miRNAs could be indirectly regulating cyclin T1 [76]. On the contrary, a very recent article from the same authors [77] shows how in activated CD4^+^ T cells miR-132 is upregulated and potentiates viral replication, probably through downregulation of MeCP2 (methyl-CpG binding protein 2, a transcriptional regulatory protein). In the same cell system (CD4^+^ T cells), Chang and coworkers have profiled the expression of cellular miRNAs and some sncRNA after HIV infection using next generation sequencing [78]. The authors used different time points after HIV-1 infection, and they found a specific miRNAs signature in the early phase of infection. Moreover, they characterized the changes in the transcriptional landscape of infected cells, due to the differential miRNA expression. Their work marks the start of a new phase in analyzing the interactions between HIV-1 and cellular sncRNAs. 

Another class of cells that are less permissive towards HIV-1 infection is peripheral blood monocytes. When differentiating into macrophages, those cells appear to downregulate expression of several miRNA, and among them, one (miR198) was identified as negative regulator of cyclin T1, a cell-cycle regulator protein. Sung and Rice in 2009 suggested that regulation of this protein could influence HIV-1 proviral expression and infection, being a crucial *Tat* cofactor. [79]. However a recent study, reviewing the literature and testing different miRNAs expression models in the monocyte/macrophage system, has found little or no expression of miR198 in primary monocytes, thus questioning the role of this miRNA in HIV-1 infection mechanisms [80]. Subsequently another report identifies overexpression of the “so-called” “anti-HIV” miRNA found in resting CD4^+^ T cells as well as in monocytes, and the authors suggest that this could be a leading mechanism in monocyte resistance [81]. Although also this report has been subsequently questioned [82], due to the use of combined miRNA inhibitors which could affect different genes, its importance remains pivotal, considering that the data were previously validated in previous studies and in a different model (CD4^+^ T cells). However, when considering the work of Specht et al. [63], we should be aware that it has not been confirmed or replicated, and thus it awaits further confirmations. 

### 3.2. miRNA Encoded by HIV-1

At current time, no general agreement is present in the field about the possibility that HIV-1 genome encodes functional miRNAs and how (if this would be the case) this fits into the existing paradigm of miRNA-based gene regulation. One of first evidences for sncRNAs encoded in HIV-1 sequences was found by Bennasser and coauthors in 2004 [83], where they hypothesized the presence of a TAR hairpin miRNA-like in HIV-1 genome. Following this paper, another confirmation from the same authors has been made in 2005 [84] where they demonstrated that in HIV-1 genome 19-bp sequences perfectly duplex and theoretically able form stem-loop structures are present. Another group independently demonstrated the presence and the activity of a miRNA-like sequence on the *nef* gene encoded by HIV-1 [85, 86]. Furthermore a recent paper reports the ability of TAR miRNA to downregulate excision repair cross-complementing rodent repair deficiency, complementation group 1 (ERCC1), and immediate early response 3 (IER3) proteins, thus blocking apoptosis in infected cells [87], so a possible function of this miRNA has been in part clarified.

However these results have been contested by Lin and Cullen in an extensive work. They used different cell lines and PBMCs from HIV-1-positive individuals assessing the presence or the absence of sncRNA with small RNA cloning strategy: almost no miRNA or siRNA in HIV-1-infected cells has been found [88]. On the contrary, some recent reports suggest the existence of HIV-1-encoded miRNA. Yeung and coworker using pyrosequencing analyzed HIV-1 latently infected human monocyte and T-cell lines, and they found numerous small ncRNAs encoded by HIV-1 [89]. A more recent study used deep sequencing to evaluate the presence of HIV-1-derived miRNA in infected cells. They found on the “plus” strand of HIV-1 genome two putative viral miRNA: one (vmiRNA-43/9175) already found in a previous work [87] in the TAR region and a new one (vmiRNA-2413) in the pol gene; moreover, they identified possible viral miRNA also on the “minus” strand. Specifically, the authors found that the 3′ end of HIV-1 genome could produce different small antisense RNA, and that they can form dsRNA with the sense transcript, inhibiting viral production through activation of the siRNA machinery. *Tat* protein has a potent SRS activity (see previous section), and probably it helps overcoming the mechanism just described that could be started by insertion of the viral genome with an upstream promoter near the 3′ ends. More interestingly, this mechanism could in theory be exploited to block HIV-1 replication, thus opening possible novel therapeutic opportunities. 

Nevertheless a very recent paper by Cullen and coauthors demonstrates instead that HIV-1 genome is neither sensitive to cellular miRNA or expressing its own miRNA. The authors using deep sequencing failed to find any putative miRNA encoded by HIV-1 in different cell-infected lines, and moreover, they did not find any cellular miRNA binding to HIV-1 genome, although multiple putative binding sequences are present [90]. We can then conclude that the existence of viral encoded miRNA is deeply controversial, needing more work to confirm its existence; if the existence confirmed, then new opportunities to conceive original therapeutic strategies to fight HIV-1 based on miRNA could arise.

## 4. Conclusion and Future Perspective

In this review we have focused on recently discovered mechanisms whereby the HIV-1 virus escapes the immune response, also by way of interactions with novel partners, i.d. HLA-C, and HLA-G.

Our knowledge about these interactions and about the role of the RNA interference mechanism in HIV-1 infection and progression is still at a nascent stage; one main question remains if whatever HIV-1 encoded miRNA exists and which role it could possibly have in the disease progression. We are wondering if

- those putative virus-encoded miRNA are linked in some yet unknown way to the HLA-C and HLA-G roles in HIV-1 infection,
- those miRNA could exert another completely different role.

If the first hypothesis (a) is true, we will confirm the existence and the functionality of HIV-1-encoded miRNA; the recent studies reported and discussed in this review seem to provide solid proofs on this possibility.

Another interesting point that we would like to raise is related to the 3′ antisense transcript from the HIV-1 genome: is this long, ncRNA a precursor of different and physiologically active miRNAs and which role may they have in the disease progress? Until now no definitive study has been able to answer this question, but we do think that it is an issue of great interest in HIV-1-infection biology, and future works will be surely consider this point.

When considering HLA-G, another question remains unanswered: is HLA-G expression upregulated in response to IL-10 increase or simply in correlation to infective state? Answering this question could be quite important not only in AIDS but also in relation to other pathologies, and simple experiments could solve this puzzle: in analogy on what has been done in tumors [91], it will be possible to examine the transcriptional response of HLA-G during stimulation with IL-10 in HIV-1 infected cells. 

The identification of novel mechanisms involved in HLA-G and HLA-C gene expression regulation trough miRNAs, able to influence HIV-infection susceptibility, virus replication, and disease progression, opens a new era for genetic hunters looking for variations in host genome capable to modulate the response to the virus, possibly helping to discover new strategies to fight HIV. The interactions within host and virus genome are probably more complex than what is known up to date, and the tuning of gene expression regulation through miRNAs represents a new genomic universe to be investigated. 

## Abbreviations

ncRNA: Non-coding RNA
tRNA: Transfer RNA
rRNA: Ribosomal RNA
snoRNA: Small nucleolar RNA
piRNA: Piwi-interacting RNA
miRNA: MicroRNA
MHC: Major histocompatibility complex
HLA: Human leukocyte antigen
KIR: Killer cell immunoglobulin-like receptor
LILRB1-2: Leukocyte immunoglobulin-like receptor
NK: Natural killer
CTL: Cytotoxic T lymphocytes
ERCC1: Excision repair cross-complementing rodent repair deficiency, complementation group 1
IER3: Immediate early response 3.
